# Real‐World Effectiveness and Safety of Tirzepatide in Type 1 Diabetes and Obesity: Impact on Glycaemia, Weight, and Cardiometabolic Risk Markers

**DOI:** 10.1111/dom.71080

**Published:** 2026-07-06

**Authors:** Simon A. Berry, Iona R. Goodman, Eleanor McNally, Jackie Elliott, Ahmed Iqbal

**Affiliations:** ^1^ Division of Clinical Medicine, School of Medicine and Population Health University of Sheffield Sheffield UK; ^2^ Department of Diabetes and Endocrinology Sheffield Teaching Hospitals NHS Foundation Trust Sheffield UK

**Keywords:** antidiabetic drug, GLP‐1 analogue, insulin resistance, obesity therapy, real‐world evidence, type 1 diabetes

## Abstract

**Aims:**

To evaluate the effectiveness and safety of tirzepatide on glycaemia, weight, and markers of cardiometabolic risk in people with type 1 diabetes and obesity.

**Materials and Methods:**

This retrospective cohort study compared 142 adults with type 1 diabetes (BMI ≥ 30 kg/m^2^) initiated on tirzepatide against 50 age‐, sex‐ and BMI‐matched controls. Outcomes included changes in HbA1c, weight, total daily dose of insulin, cardiometabolic markers, and safety data including hospitalisations over 12 months.

**Results:**

Participants (mean age 42.8 years, BMI 37.4 kg/m^2^, HbA1c 64.3 mmol/mol, 32% male) were followed up for a median of 365 days. The median tirzepatide dose (72.4%) was 5 mg weekly. Compared to controls, the tirzepatide group achieved significant mean reductions in: HbA1c (6.5 mmol/mol [95% CI 3.8–9.1], *p* < 0.001) (0.6% [0.4–0.8]), weight (13.4 kg [11.0, 15.8], *p* < 0.001) and total daily insulin dose (29.9 units [16.9, 42.9], *p* < 0.001). Significant improvements were observed in systolic blood pressure (12.6 mmHg, *p* < 0.001), diastolic blood pressure (4.1 mmHg, *p* = 0.034), total cholesterol (0.8 mmol/L, *p* < 0.001), non‐HDL cholesterol (0.6 mmol/L, *p* < 0.001) and triglycerides (0.7 mmol/L, *p* < 0.001) compared to controls. While side effects were common, 89.4% continued on tirzepatide at 1 year. There was no between‐group difference in severe hypoglycaemia, ketoacidosis nor hospitalisation rates.

**Conclusions:**

In a real‐world setting, tirzepatide significantly improves glycaemia, weight, and markers of cardiometabolic risk in people with type 1 diabetes and obesity with a reassuring safety profile. These findings warrant large‐scale RCTs to evaluate long‐term cardiovascular outcomes in this high‐risk population.

**Abbreviations**
ACR: albumin‐creatinine ratio
CGM: continuous glucose monitoring
CV: coefficient of variation
DBP: diastolic blood pressure
EHR: electronic health records
GLP‐1RA: GLP1 receptor agonists
GMI: glycaemic management index
HCL: hybrid closed loop
IMD: index of multiple deprivation
MDI: multiple daily injection
NHS: national health service
RCT: randomised controlled trial
SBP: systolic blood pressure
TAR: time above range
TBR: time below range
TDD: total daily dose of insulin
TIR: time in range
TITR: time in tight range

## 1 Introduction

Intensive insulin therapy has been demonstrated to unequivocally reduce the risk of microvascular and macrovascular complications in type 1 diabetes [1]. However, cardiovascular disease remains the predominant cause of death in these individuals [2]. Despite improvements in recent decades, life expectancy remains around 10 years lower in people with type 1 diabetes than in those without [2]. The seminal DCCT and EDIC studies demonstrate that insulin‐induced weight gain offsets the cardiovascular benefits achieved by intensive insulin therapy [1]. The development of insulin resistance in type 1 diabetes is multifactorial and part of a metabolic syndrome that has an estimated 40% prevalence after 9 years of diabetes duration [3, 4]. The prevalence of obesity in people with type 1 diabetes is rising, thereby augmenting insulin resistance, and increasing the risk of macrovascular complications and overall mortality [1, 3, 5]. This provides a potential target for reducing cardiovascular complications and narrowing the life expectancy gap.

GLP1 receptor agonists (GLP‐1RAs) have transformed the management of type 2 diabetes by reducing dysglycaemia, promoting weight loss, increasing insulin sensitivity, improving steatotic liver disease, and providing cardio‐renal protection [3, 6], whilst providing additional benefits to traditional cardiovascular risk factors such as dyslipidaemia and hypertension [7, 8]. People with type 1 diabetes were excluded from the original large cardiovascular outcome studies with GLP‐1RAs [3]. The ADJUNCT‐ONE and ADJUNCT‐TWO randomised controlled trials (RCTs) demonstrated that liraglutide provided modest glycaemic and weight‐loss benefits in type 1 diabetes, yet potential concerns were raised in relation to increased rates of hypoglycaemia and ketosis. Consequently, the risk‐benefit profile was deemed insufficient for regulatory approval [9, 10]. However, off‐label use of GLP‐1RAs is common [5]. Dual GLP‐1/GIP receptor agonists such as tirzepatide have now been developed on the premise that GIP signalling amplifies the therapeutic benefits of GLP‐1 receptor activation. Tirzepatide has been demonstrated to be superior in terms of weight loss and glycaemic benefits when compared to semaglutide in type 2 diabetes [11].

An important question is whether dual GLP‐1/GIP agonists can reduce insulin resistance and mitigate cardiometabolic risk in type 1 diabetes. In the absence of large‐scale Phase 3 RCTs, we aimed to address this evidence gap by conducting a retrospective comparative cohort study. We investigated clinical outcomes over a 12‐month period following tirzepatide initiation, specifically examining changes in HbA1c, weight, exogenous insulin requirements, continuous glucose monitoring (CGM) metrics, cardiometabolic risk markers such as cholesterol and blood pressure, and safety and adverse event data.

## 2 Materials and Methods

### 2.1 Study Design and Participants

A single‐centre retrospective matched‐cohort study was conducted at Sheffield Teaching Hospitals NHS Foundation Trust (United Kingdom). The project was approved as an institutional case notes review (Reference 12260). All people with type 1 diabetes prescribed tirzepatide between March 2024 and June 2025 as part of routine clinical practice were identified from electronic health (EHR) and pharmacy records. Fifty age‐, sex‐ and BMI‐matched controls with type 1 diabetes and obesity were identified from the same clinical records. This sample size was chosen pragmatically to balance statistical power with data collection burden and data granularity. There were no exclusion criteria for controls, with other GLP‐1RA use allowed.

### 2.2 Intervention

Tirzepatide was prescribed on a case‐by‐case basis by individual clinicians with no fixed protocol. General departmental guidance suggested a maximum maintenance dose of 5 mg once weekly (due to cost), except for exceptional circumstances, and a minimum 4‐week titration interval. At initiation, individuals were advised to adjust their insulin doses dependent upon glucose results. Patients were counselled on common gastrointestinal side effects, and more serious potential adverse effects including worsening of diabetic retinopathy, acute pancreatitis, gallstones, and bowel obstruction.

### 2.3 Data Collection

Clinical data from EHR (weight, insulin dose, and blood pressure), laboratory results (blood and urine), and 14‐day CGM metrics (Dexcom Clarity, Libreview, and Glooko) were extracted at 3‐month intervals for up to 12 months. Adverse events were extracted from clinical notes. Hospitalisations included all emergency department attendances, hospital ambulatory care attendances, and inpatient stays, regardless of whether scheduled or unscheduled. An episode of severe hypoglycaemia was defined as hypoglycaemia leading to acute severe cognitive impairment requiring external assistance for recovery [12]. Retinopathy status was extracted from National Health Service (NHS) diabetes eye screening records, in which there is independent grading by a minimum of two assessors. Under the NHS features‐based grading framework, diabetic retinopathy is stratified into four tiers: no retinopathy (R0), background retinopathy (R1), pre‐proliferative retinopathy (R2), and proliferative retinopathy (R3) [13]. Concurrently, maculopathy is classified as either no maculopathy (M0) or referable diabetic maculopathy (M1). In this study, progression of retinopathy was defined as worsening by one or more grades. End‐of‐follow‐up data indicates the last available data point within the 12‐month follow‐up period.

### 2.4 Statistical Analysis

The pre‐specified primary outcomes were longitudinal changes in HbA1c, weight, total daily dose of insulin, CGM metrics, metabolic metrics, and safety data. To isolate the effect of tirzepatide, participants were excluded from specific dependent analyses if there was a change in insulin mode of delivery (such as multiple daily injection to hybrid closed loop for HbA1c and CGM metrics) or modification of relevant medications, such as antihypertensives (for blood pressure, eGFR, and urinary ACR) or statins (lipid parameters) during the follow‐up period.

Analyses were conducted in R Studio (v2025.09.2 + 418, Posit, Boston, MA). Parametric data were analysed using paired and independent *t*‐tests, and non‐parametric data were analysed using Wilcoxon‐ranked sign tests and Mann–Whitney *U* tests. Hospitalisation and adverse event rates and changes in retinopathy status were analysed via an intention‐to‐treat approach. For hospitalisation rates, a Quade test (rank‐based ANCOVA) was applied to account for differences in baseline rates. Incomplete data pairs were excluded from individual analyses. Pre‐specified exploratory subgroup analyses compared hybrid closed loop users (HCL) versus multiple daily injections (MDI) users, as well as GLP‐1 agonist‐naïve individuals versus those switched directly from other GLP‐1 therapies.

## 3 Results

### 3.1 Baseline Characteristics

The 142 participants with type 1 diabetes initiated on tirzepatide during the study period were identified, with 50 matched controls. Baseline demographics and clinical markers were generally well‐balanced between groups (Tables 1 and S1). However, the tirzepatide group had a higher baseline total daily dose of insulin (TDD) compared to controls (89.6 ± 57.6 units versus 62.4 ± 46.7 units). Additionally, a higher proportion of participants in the tirzepatide group utilised HCL systems (42.3% vs. 30.0%).

**TABLE 1:** Baseline characteristics.

	Controls (*n* = 50)	Tirzepatide (*n* = 142)	Overall (*n* = 192)
Age (years)	43.1 (15.4)	42.7 (14.6)	42.8 (14.8)
Diabetes duration (years)	24.0 (15.2)	21.3 (13.9)	22.0 (14.3)
IMD (decile)	4.8 (3.2)	4.8 (3.1)	4.8 (3.1)
Weight (kg)	103.0 (14.1)	109.0 (20.0)	107.4 (18.8)
Body mass index (kg/m2)	36.7 (4.0)	37.7 (5.4)	37.4 (5.1)
Systolic blood pressure (mmHg) (*n* = 189)	134.6 (15.2)	137.2 (18.1)	136.5 (17.4)
Diastolic blood pressure (mmHg) (*n* = 189)	80.8 (11.9)	81.4 (11.0)	81.3 (11.2)
HbA1c (mmol/mol) (*n* = 190)	64.2 (12.7)	64.4 (12.5)	64.3 (12.6)
HbA1c (%)	8.0 (1.2)	8.0 (1.1)	8.0 (1.2)
Total cholesterol (mmol/L) (*n* = 189)	4.4 (0.9)	4.4 (1.0)	4.4 (1.0)
Non‐HDL cholesterol (mmol/L) (*n* = 189)	2.9 (0.9)	3.1 (1.0)	3.0 (1.0)
Triglycerides (mmol/L) (*n* = 189)	1.2 (0.5)	1.7 (1.6)	1.6 (1.4)
ALT (IU/L)	21.1 (11.7)	23.6 (16.8)	22.9 (15.6)
Total daily dose of insulin (units) (*n* = 105)	62.4 (46.7)	89.6 (57.6)	85.7 (56.8)
Urinary ACR (mg/mmol) (*n* = 173)	0.5 [0.0–2.0]	0.6 [0.0–1.5]	0.6 [0.0–1.6]
eGFR (mL/min/1.73m^2^)	90.0 [83.0–90.0]	90.0 [78.0–90.0]	90.0 [79.0–90.0]
Sex (Male)	16 (32%)	46 (32.4%)	62 (32%)
Ethnicity			
White British	43 (86.0%)	124 (87.3%)	167 (87.0%)
White Other	2 (4.0%)	3 (2.1%)	5 (2.6%)
Asian	0 (0.0%)	5 (3.5%)	5 (2.6%)
Black	2 (4.0%)	4 (2.8%)	6 (3.1%)
Mixed	3 (6.0%)	2 (1.4%)	5 (2.6%)
Not known	0 (0.0%)	4 (2.8%)	4 (2.1%)
Concomitant medication			
Metformin	10 (20.0%)	39 (27.5%)	49 (25.5%)
SGLT2i	0 (0.0%)	1 (0.7%)	1 (0.5%)
Cholesterol‐lowering medication	29 (58.0%)	88 (62.0%)	117 (60.9%)
Antihypertensive medication	22 (44.0%)	66 (46.5%)	88 (45.8%)
Other GLP‐1 agonists	3 (6.0%)	NA	NA
Mode of insulin delivery			
Multiple daily injection	34 (68.0%)	74 (52.1%)	108 (56.3%)
Hybrid closed loop pump therapy	15 (30.0%)	60 (42.3%)	75 (39.1%)
Open loop pump therapy	1 (2.0%)	6 (4.2%)	7 (3.6%)
Mixed insulin	0 (0.0%)	2 (1.4%)	2 (1.0%)
Direct switch from another GLP‐1 agonist	NA	30 (21.1%)	NA
Comorbidities			
MASLD	2 (4.0%)	15 (10.6%)	17 (8.9%)
Hypertension	26 (52.0%)	70 (49.3%)	96 (50.0%)
Cardiovascular disease	5 (10.0%)	19 (13.4%)	24 (12.5%)
Anxiety	5 (10.0%)	31 (21.8%)	36 (18.8%)
Depression	6 (12.0%)	41 (28.9%)	47 (24.5%)
Gastroparesis	1 (2.0%)	3 (2.1%)	4 (2.1%)
Painful peripheral neuropathy	0 (0.0%)	17 (12.0%)	17 (8.9%)
Peripheral vascular disease	2 (4.0%)	2 (1.4%)	4 (2.1%)
Previous stroke	3 (6.0%)	7 (4.9%)	10 (5.2%)
Retinopathy status (*n* = 187)			
R0—no retinopathy	9 (18.8%)	30 (21.6%)	39 (20.9%)
R1—background retinopathy	23 (47.9%)	60 (43.2%)	83 (44.4%)
R2—pre‐proliferative retinopathy	6 (12.5%)	24 (17.3%)	30 (16.0%)
R3—proliferative retinopathy	10 (20.8%)	25 (18.0%)	35 (18.7%)
Maculopathy Status (*n* = 186)			
M0—no maculopathy	38 (80.9%)	107 (77.0%)	145 (78.0%)
M1—referable maculopathy present	9 (19.1%)	32 (23.0%)	41 (22.0%)

*Note:* 142 people with type 1 diabetes initiated on tirzepatide were identified, with 50 age‐, sex‐ and BMI‐matched controls. Continuous variables are described as mean (SD) if parametric, and median [IQR1‐IQR3] if non‐parametric. Categorical variables are described as *n* (% proportion). *N* numbers are given for variables with incomplete baseline data. Retinopathy and maculopathy gradings as per NHS diabetic eye screening programme [13].

Abbreviations: ACR, albumin creatinine ratio; ALT, alanine aminotransferase; eGFR, estimated glomerular filtration rate; GLP‐1, glucagon‐like peptide‐1; IMD, Index of Multiple Deprivation; MASLD, metabolic dysfunction‐associated steatotic liver disease; SGLT2i, sodium/glucose co‐transporter 2 inhibitor.

Median follow‐up was 365 days (IQR 261–365) for the tirzepatide group, while all controls had 365 days complete data. At end of follow‐up, 127 of the 142 participants (89.4%) remained on tirzepatide. Most participants were maintained on 5 mg once weekly (72.4%), followed by 2.5 mg (15%), 7.5 mg (6.3%), 10 mg (4.7%) and 15 mg (1.6%). Thirty individuals (21.1%) in the tirzepatide group had switched directly from another GLP1‐RA, predominantly due to international supply issues.

### 3.2 Haemoglobin A1c

**FIGURE 1:**
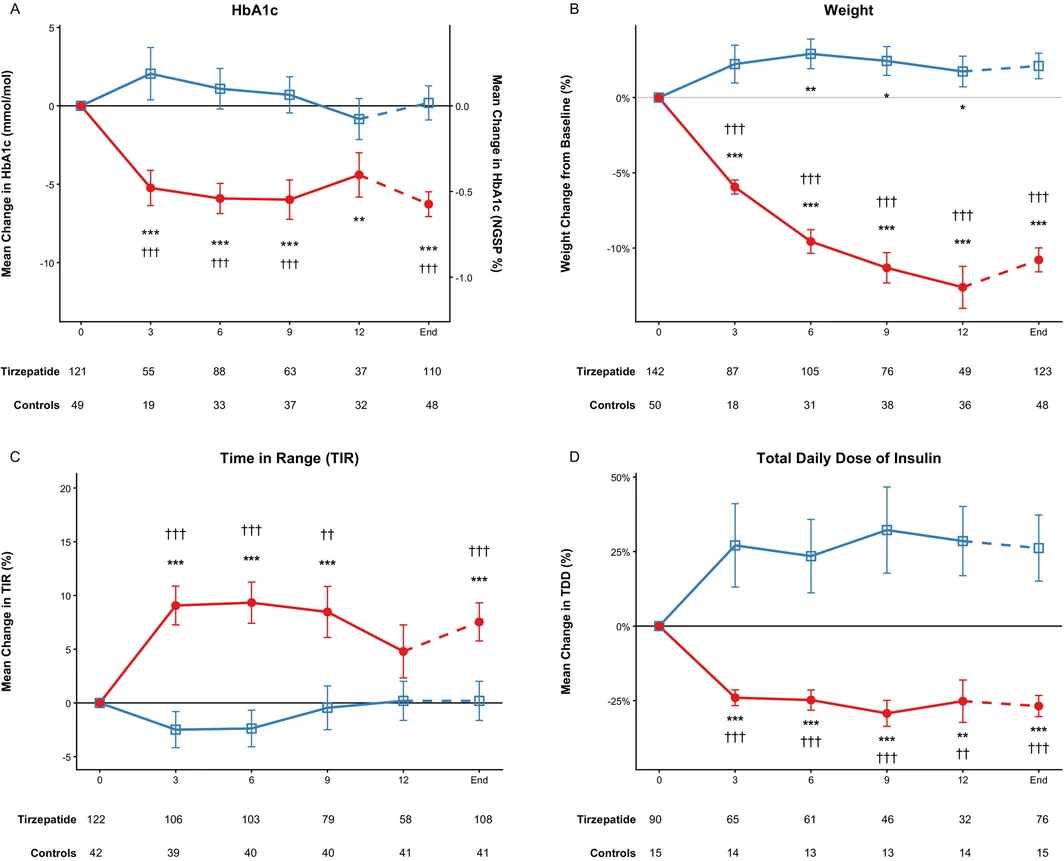
Longitudinal glycaemic and weight outcomes over follow‐up period (0, 3, 6, 9 and 12 months, and end‐of follow‐up). Data represented as mean ± SE for (A) HbA1c (mmol/mol, NGSP %), (B) percentage weight change, (C) percentage change in time in range (TIR), and (D) percentage change in total daily dose of insulin. Tirzepatide is represented by red lines with filled circles; controls are represented by blue lines with open squares. Significance relative to baseline is indicated by asterisks (* *p* < 0.05, **p < 0.01, ***p < 0.001; paired *t*‐test). Significance between groups is indicated by daggers († *p* < 0.05, †† *p* < 0.01, ††† *p* < 0.001; independent samples *t*‐test). Sample sizes (*n*) for each group at each time point are provided in the tables below the respective axes.

Following exclusion of participants with changes in insulin delivery mode and missing data, the final analysis included 110 participants in the tirzepatide group and 48 in the control group. At the end of follow‐up, the tirzepatide group demonstrated a significant mean reduction in HbA1c of 6.5 mmol/mol ([95% CI 3.8–9.1], *p* < 0.001) (0.59% [0.35%–0.83%]) compared to the control group (Figure 1A). Achievement of glycaemic targets was significantly higher in the tirzepatide group; specifically, more participants attained HbA1c levels < 64 mmol/mol (8.0%) (76.6% vs. 56.2%, *p* = 0.017), < 58 mmol/mol (< 7.5%) (51.4% vs. 29.2%, *p* = 0.016), and < 53 mmol/mol (< 7.0%) (36.0% vs. 14.6%, *p* = 0.011) compared to controls.

Stratification by baseline HbA1c demonstrated significant reductions across all HbA1c quartiles within the tirzepatide group, with the magnitude of effect increasing with higher baseline values. Mean reductions by quartile were: Q1 2.8 mmol/mol (mean at baseline 50.2 mmol/mol, *p* = 0.019), Q2 3.8 mmol/mol (59.8 mmol/mol, *p* = 0.006), Q3 6.5 mmol/mol (66.8 mmol/mol, *p* < 0.001) and Q4 12.3 mmol/mol (80.0 mmol/mol, *p* < 0.001).

### 3.3 Weight

The mean reduction in weight at end of follow‐up was 13.4 kg with tirzepatide (−11.5 kg, *n* = 123) compared to controls (+1.9 kg, *n* = 48, *p* < 0.001) (Figure 1B). The mean percentage reduction in weight was 10.8% in the tirzepatide group. Significantly greater proportions of patients on tirzepatide achieved weight loss of ≥ 5% (71.5% vs. 8.3%, *p* < 0.001) and ≥ 10% (44.7% vs. 2.1%, *p* < 0.001) than controls. There was no significant correlation between change in weight and change in HbA1c (*r* = −0.06, *p* = 0.519).

### 3.4 Metabolic Parameters

**FIGURE 2:**
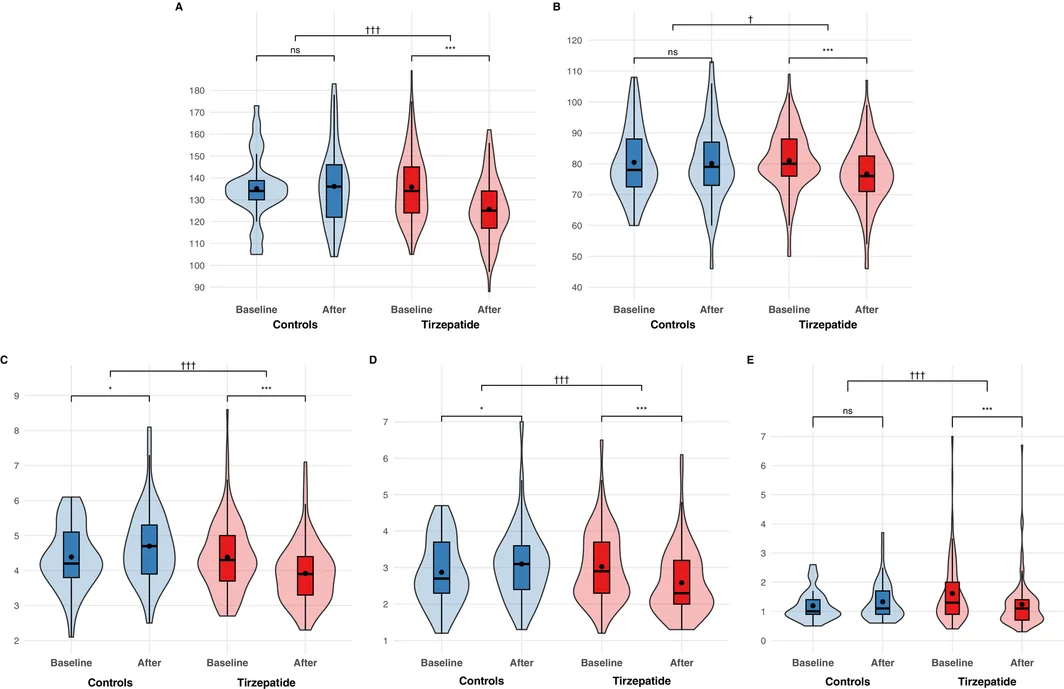
Metabolic parameters before and after initiation of tirzepatide versus controls (A) Systolic Blood Pressure (mmHg), (B) Diastolic Blood Pressure (mmHg), (C) Total Serum Cholesterol (mmol/L), (D) non‐HDL Cholesterol (mmol/L), (E) Serum Triglycerides (mmol/L). Sample sizes were *n* = 113 (tirzepatide) and *n* = 45 (controls) for blood pressure parameters, and *n* = 96 (tirzepatide) and *n* = 45 (control) for lipid parameters. Data are presented as violin plots showing underlying distribution, with box plots indicating median and IQR, and black circles as the mean. Asterisks denote significant within‐group changes from baseline (* *p* < 0.05, **p < 0.01, ***p < 0.001; paired *t*‐tests) and daggers denote significances between groups after follow‐up († *p* < 0.05, †† *p* < 0.01, ††† *p* < 0.001; independent samples *t*‐tests).

Significant reductions in systolic blood pressure (SBP) (12.6 mmHg [6.2–19.1], *p* < 0.001) and diastolic blood pressure (DBP) (4.1 mmHg [0.3–7.9], *p* = 0.034) were observed in the tirzepatide group (*n* = 113) compared to controls (*n* = 45) (Figure 2A,B). Those on tirzepatide more frequently achieved SBP targets of < 140 mmHg (85.2% vs 62.2%, *p* = 0.001) and < 130 mmHg (61.7% vs. 35.6%, *p* = 0.003), but DBP target attainment (< 90 and < 80 mmHg) did not differ significantly.

There were significant between‐group reductions in total cholesterol (0.8 mmol/L [0.4–1.1], *p* < 0.001), non‐HDL cholesterol (0.6 mmol/L [0.4–0.9], *p* < 0.001) and triglycerides (0.7 mmol/L [0.3–1], *p* < 0.001) with tirzepatide (*n* = 96) versus controls (*n* = 45) (Figure 2C–E). Mean baseline ALT concentrations were similar between the tirzepatide (22.9 IU/L, *n* = 109) and control groups (21.2 IU/L, *n* = 47), with both falling within the normal reference range. At follow‐up, no statistically significant between‐group difference was observed (−1.7 IU/L, 95% CI −2.1 to 5.5; *p* = 0.380).

From baseline values in the normal laboratory reference ranges, there was no significant difference in eGFR during follow‐up between tirzepatide (*n* = 105) and controls (*n* = 46) (0 mL/min/1.73m^2^, *n* = 105 vs. 46, *p* = 0.113), nor in urinary ACR (−0.05 mg/mmol, *n* = 74 vs. 34, *p* = 0.063).

### 3.5 Insulin Doses

Following tirzepatide initiation (*n* = 76), TDD decreased by 24.5 units (26.8%) from baseline ([16.3–32.7], *p* < 0.001), representing a 26.8% decrease. This represented a 29.9 unit ([16.9–42.9], *p* < 0.001) reduction compared to controls (*n* = 15) (Figure 1D). The weight‐adjusted insulin dose significantly reduced from 0.843 to 0.668 units/kg in the tirzepatide group (*p* < 0.001, *n* = 75), whereas there was no significant change in the control group (0.614–0.640 units/kg, *p* = 0.633, *n* = 15). The TDD reduction comprised significant decreases in both bolus (13.2 units [8.4–18.1], 31.0%, *n* = 74, *p* < 0.001) and basal insulin (12.9 units [8.6–17.2], 20.8%, *n* = 114, *p* < 0.001), with a proportionally greater reduction in bolus insulin. There was a significant (*p* = 0.006) but weak correlation between reduction in weight and reduction in TDD (*r* = 0.32, *n* = 75).

### 3.6 CGM Metrics

**FIGURE 3:**
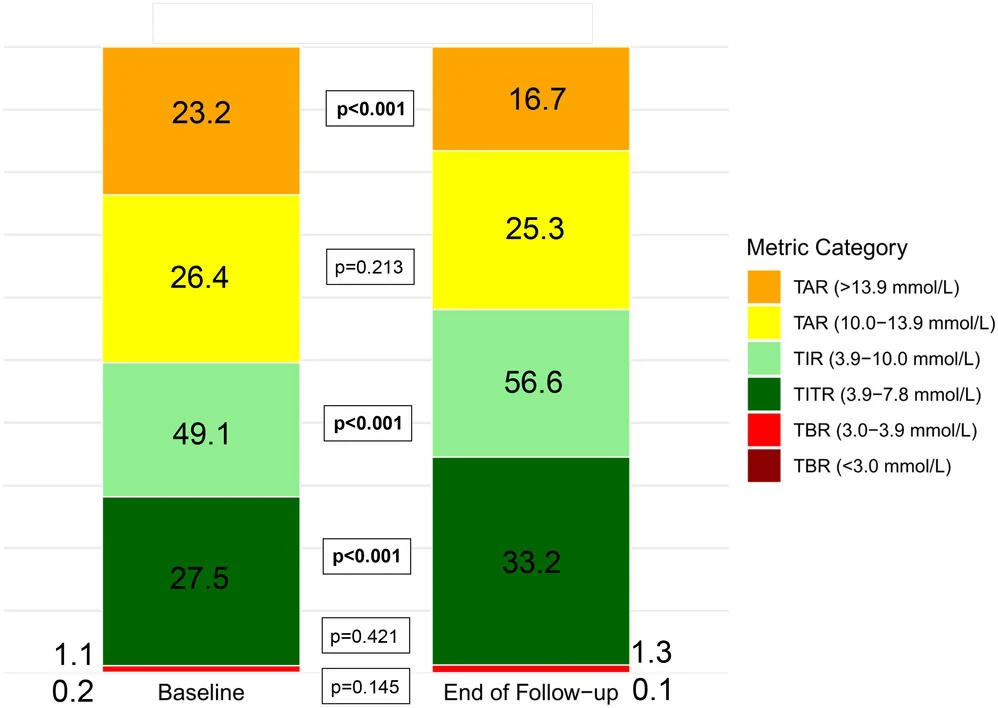
Ambulatory glucose profile (AGP) metrics in the tirzepatide cohort at baseline and end of follow‐up (*n* = 108). Data represent mean glycaemic metrics before tirzepatide initiation and at the end of the follow‐up period. *p* values were calculated using paired *t*‐tests. Time Below Range (TBR): Dark red (< 3.0 mmol/L); Red (3.0–3.9 mmol/L). Time in Range (TIR): Dark green (TITR: 3.9–7.8 mmol/L); Light green (TIR: 3.9–10.0 mmol/L). Time Above Range (TAR): Yellow (10.1–13.9 mmol/L); Orange (> 13.9 mmol/L). Both TBR variables and TAR > 13.9 mmol/L were non‐parametric in distribution. Statistical analyses for these variables were repeated with Wilcoxon Signed Rank Tests with the same levels of significance observed.

Paired CGM data were available for 41 controls and 108 in the tirzepatide group. Within the tirzepatide group, from baseline to end of follow‐up, significant improvements were seen in time in range (TIR) (7.5% [4.0–11.1], *p* < 0.001), time in tight range (TITR) (3.9–7.8 mmol/L) (5.7% [2.4–8.0], *p* < 0.001), and time above range (TAR) > 13.9 mmol/L (−6.5% [−10.1, −2.0], *p* < 0.001). These were accompanied by significant changes in mean sensor glucose (−0.9 mmol/L [−1.3, −0.4], *p* < 0.001), glycaemic management index (GMI) (−2.9 mmol/mol [−4.5, −1.3] *p* < 0.001), coefficient of variation (CV) (−1.6% [−2.7, −0.5], *p* = 0.004) and SD (−0.4 [−0.5, −0.2], *p* < 0.001) (Figure 3). There were no significant changes in TAR 10.0–13.9 mmol/L (−1.1% [−2.9, 0.7], *p* = 0.213), time below range (TBR) < 3.0 mmol/L (−0.1% [−0.2–0.0], *p* = 0.145) nor TBR 3.0–3.9 mmol/L (0.2% [−0.3–0.7], *p* = 0.165).

When compared to controls, only the increases in TIR (7.3%, [2.3–12.4], *p* = 0.005), TITR (5.2%, [1–9.4], *p* = 0.016) and the decrease in mean sensor glucose (0.8 mmol/L, [0.2–1.4], *p* = 0.009) remained statistically significant (Figure 1C, Table S2).

### 3.7 Safety Data

**FIGURE 4:**
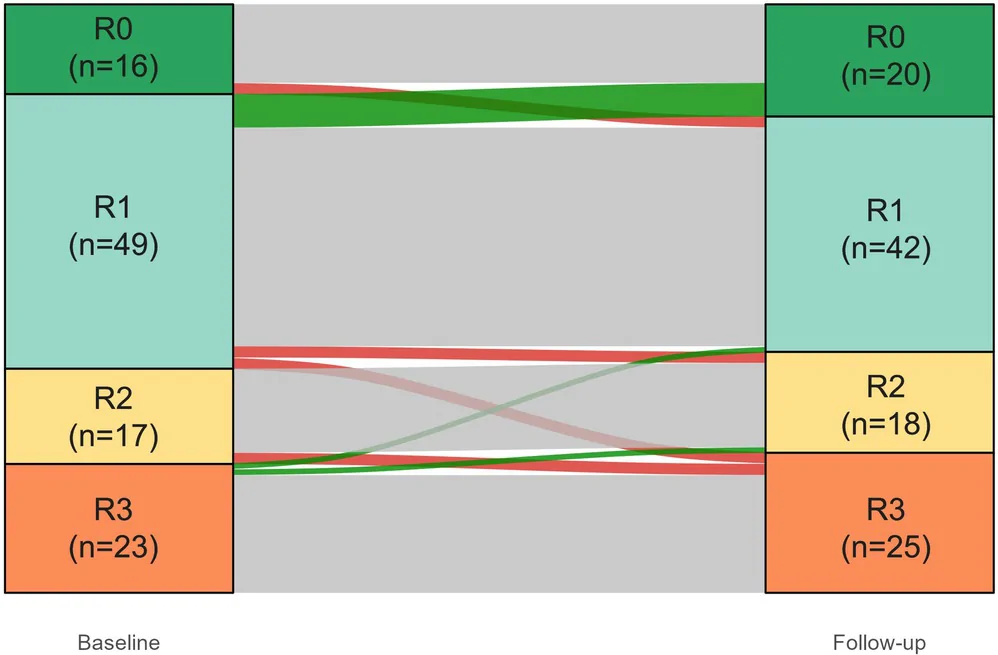
A Sankey diagram displaying change in retinopathy status in the tirzepatide group between baseline and end of follow‐up (*n* = 105). Green lines represent improvement, red lines represent deterioration and grey lines represent no change.

Forty participants in the tirzepatide cohort reported an adverse effect (28.2%). The most common adverse effect was nausea or vomiting (19.7%), followed by abdominal pain (8.5%), diarrhoea (4.9%), burping (2.8%), gallstones (1.4%), and a change in menstrual cycle (0.7%). No cases of pancreatitis, gastroparesis nor bowel obstruction were observed. Adverse events were less likely in males than females (OR 0.34 [0.13–0.81], *p* = 0.021). Annualised hospitalisation rate significantly decreased in the tirzepatide group (0.77 per participant per year to 0.43, *p* = 0.001), whereas in the control group there was no change (0.40–0.42, *p* = 0.83). There was no significant difference in the change in hospitalisation rates between the two groups (*p* = 0.18). Rates of diabetic ketoacidosis did not significantly differ between the tirzepatide group (0.04 per patient per year) and the control group (0.0, *p* = 0.182). Rates of severe hypoglycaemia also did not significantly differ between the tirzepatide (0.01 per patient per year) and the control groups (0.00, *p* = 0.405). Progression of retinopathy occurred in 8 individuals in the tirzepatide group (7.6%, *n* = 105 with paired data available) and 5 in the control group (10.4%, *n* = 48 with paired data available) (*p* = 0.792) (Figure 4).

### 3.8 HCL Subgroup Analysis

Significant improvements were seen in HbA1c, weight, TDD, TITR, TIR, TAR > 13.9 mmol/L, mean sensor glucose, and SD in MDI users in the tirzepatide cohort (Table S3). In addition to all the parameters that improved in the MDI group, significant improvements were seen in those using HCL in TAR 10.0–13.9 mmol/L and CV.

At the end of follow‐up, HCL users in the tirzepatide group achieved a mean HbA1c of 53.2 mmol/mol (±SD 8.1) (7.0% ± 0.7%) compared to 57 mmol/mol (±7.3) (7.4% ± 0.7%) in HCL users in the control group, despite the tirzepatide group having a higher baseline (60.5 vs. 57.5 mmol/mol) (Table S4). Improvements in HbA1c were statistically significant in two different HCL systems (Table S5). The tirzepatide group achieved a numerically lower CV (33.5% vs. 35.2%) and SD (3.3 vs. 3.4) compared to controls.

### 3.9 GLP‐1RA Subgroup Analysis

Participants directly switching from other GLP‐1RAs achieved significant reductions in HbA1c (6.3 mmol/mol [2.3–10.3], *p* < 0.003), weight (8.0 kg [4.6–11.3], *p* < 0.001), and total daily bolus dose (8.5 units [1.8–15.2], *p* = 0.016) (Table S6). No other significant changes were observed in the context of small numbers (*n* = 30). The magnitude of HbA1c and TDD reduction was comparable with those who were GLP‐1RA naïve, but weight loss was lower (8.0 vs. 12.7 kg).

## 4 Discussion

Our findings suggest that tirzepatide represents a promising therapeutic adjunct for individuals with type 1 diabetes and obesity, delivering a mean weight loss exceeding 10% alongside significant HbA1c reductions and improvements in TIR. Decreased daily insulin requirements, coupled with favourable improvements in cardiometabolic risk markers such as blood pressure and lipid profiles, indicate a mitigation of exogenous insulin‐driven insulin resistance. These benefits were achieved safely without increasing the risk of diabetic ketoacidosis, severe hypoglycaemia, nor early worsening of diabetic retinopathy. By addressing these surrogate markers, these real‐world data suggest that tirzepatide offers a potential pathway to narrowing the cardiovascular risk and life expectancy gap that remains a critical unmet need in type 1 diabetes management.

The observed improvements in glycaemia and weight align with other published data on the use of tirzepatide in type 1 diabetes. The TIRTLE1 study, the only published RCT to date as far as we are aware, reported an 8.8% weight reduction, 0.4% HbA1c reduction, and 35.1% decrease in insulin requirements over 12 weeks in 24 adults with type 1 diabetes [14]. While other real‐world studies with longer follow‐up have shown more pronounced HbA1c reduction (0.5%–0.9%) and weight reductions (12.2%–23.4%) [15, 16, 17, 18], these discrepancies likely reflect the use of cohorts using higher doses compared to our cohort and TIRTLE1, which predominantly used 5 mg weekly. Given that cost may pose a significant barrier to adoption, our findings demonstrate that even lower dose tirzepatide yields substantial glycaemic benefits and benefits in cardiometabolic risk markers. Interestingly, in our study, there was no significant correlation between HbA1c change and weight change in our results, similar to the heterogeneity also observed in type 2 diabetes [19].

While GLP1‐RAs have been explored in type 1 diabetes for years, regulatory approval has been hindered by the marginal risk–benefit ratios seen in early studies. Early RCTs, such as ADJUNCT ONE and TWO, including those people with type 1 diabetes and a BMI ≥ 20 kg/m^2^, reported only modest glycaemic and weight benefits alongside concerns regarding increased risk of ketosis and hypoglycaemia [9, 10]. While pooled analyses using semaglutide, a newer agent, demonstrate greater efficacy, results regarding insulin dose reduction remain inconsistent [20]. Tirzepatide has been shown to outperform semaglutide in type 2 diabetes, by enhancing insulin sensitivity and reducing HbA1c via GIP‐mediated weight‐independent mechanisms [11, 21]. Our findings provide some support for this, by showing numerical improvements in HbA1c, weight and insulin requirements that exceed those of studies using older agents. In our exploratory subgroup analysis assessing those who switched from another GLP1‐RA, significant additional reductions in HbA1c, weight and total daily dose of bolus insulin were observed despite small numbers. These results raise important questions for future research. First, is tirzepatide superior to semaglutide as a therapeutic adjunct in type 1 diabetes, and second, does targeting specific subgroups, such as those with diagnosed obesity or insulin resistance, rather than using broader populations such as those used in the ADJUNCT trials, achieve greater therapeutic results?

The development of HCL systems has transformed type 1 diabetes management, raising questions about the need for therapeutic adjuncts [22]. A recent RCT investigating semaglutide as an adjunct to automated insulin delivery found that semaglutide increased TIR by 4.8% without any increases in TBR, alongside weight loss of 5.3 kg [22]. The findings of our exploratory sub analyses further suggest benefits for tirzepatide alongside these systems. HCL users using tirzepatide in our study achieved a mean weight loss of 10.7 kg, a 6.8% increase in TIR, a 4.3% increase in TITR, an HbA1c reduction of 7.3 mmol/mol, and significant reductions in glycaemic variability. These data suggest adjunctive therapy remains an important future direction of research. Furthermore, adjunctive therapies including GLP‐1Ras and GLP1‐GIP co‐agonists may present a potential strategy to facilitate or aid the transition towards fully closed‐loop systems [23, 24]. By suppressing postprandial glucagon secretion and slowing gastric emptying, these therapies could flatten meal‐related glucose excursions, theoretically reducing the need for user‐initiated meal boluses, including in individuals without obesity.

In type 1 diabetes, exogenous insulin‐induced insulin resistance is a known driver of microvascular and macrovascular complications, as well as overall cardiovascular risk [1, 4]. While tirzepatide has already been demonstrated to improve insulin resistance and cardiometabolic risk markers in type 2 diabetes [7, 8], and has established cardiovascular non‐inferiority to dulaglutide in the SURPASS‐CVOT study, the benefits in type 1 diabetes require further investigation [6]. Our observed decrease in daily insulin doses indicates at least partial mitigation of insulin resistance, in concordance with the results of the TIRTLE1 study [14], and may explain the improvements in cardiometabolic risk factors such as lipid profile and blood pressure observed. These metabolic benefits have also been demonstrated in other real‐world data [16] but they remain surrogate markers for long‐term health. Two recently published emulation studies suggest that these improvements translate into hard outcomes. Interrogation of health service data via the TriNetX database demonstrated a marked reduction in all‐cause mortality (HR 0.18), albeit only over 2 years follow‐up [25]. A further study demonstrated significantly lower risks of major adverse cardiovascular events and end‐stage kidney disease with GLP‐1RAs in type 1 diabetes [26]. However, large scale RCTs over a longer follow‐up are required to definitively answer this question.

Safety concerns, particularly regarding hypoglycaemia and ketoacidosis, have historically been potential barriers to GLP1‐RA use in type 1 diabetes [9, 10]. However, we observed no increase in TBR, and no increased risk of severe hypoglycaemia nor diabetic ketoacidosis, with pooled analyses of GLP1RA use in type 1 diabetes further suggesting these risks may be less pronounced than previously thought [27]. However, it remains prudent for clinicians to avoid overly acute insulin dose reductions upon initiating tirzepatide, while simultaneously reinforcing sick day rules and ketone monitoring. While gastrointestinal side effects were common, as expected [28], they were generally well‐tolerated, with ~90% of participants continuing on tirzepatide at follow‐up. There were no cases of pancreatitis, in contrast to other studies which show a small increased risk of acute pancreatitis with GLP1‐RAs [29]. Furthermore, despite concerns published in literature regarding the early progression of diabetic retinopathy [30], we found no statistically significant difference between groups, with rates numerically lower in the tirzepatide cohort. Overall, these findings provide a reassuring safety profile.

## 5 Strengths and Limitations

The primary strength of this study is its robust real‐world dataset including 142 individuals with type 1 diabetes treated with tirzepatide and 50 matched controls. With no strict inclusion criteria as in RCTs, our cohort reflects a socioeconomically diverse population, enhancing the generalisability of our findings. A significant methodological strength was the thorough review of clinical records to exclude individuals with treatment changes from specific dependent individual analyses, for example, those with a change of insulin modality were excluded from glycaemic analyses. This helped to minimise confounding from real‐world treatment changes. Furthermore, the use of a real‐world cohort enabled observation of clinical heterogeneity such as the varied initiation and titration strategies that reflect actual clinical practice rather than study protocols which can be rigid and unfeasible to reproduce.

The retrospective single‐centre design also conveys limitations. Results may be affected by missing data, and reliance on clinical notes may have led to underreporting of mild‐to‐moderate adverse events. Additionally, while total daily insulin dose served as a pragmatic surrogate for insulin resistance, the lack of waist‐to‐hip ratio data meant we could not calculate more formal metrics like estimated glucose disposal rate.

At baseline, the tirzepatide group exhibited higher insulin requirements and hospitalisation rates than controls, suggesting a possible greater burden of comorbidity that prompted treatment initiation. While the subsequent reduction in TDD for tirzepatide in contrast to the increase in TDD in controls supports clinical efficacy, these results could also reflect regression to the mean. Improvements across a range of cardiometabolic risk markers and weight support a true therapeutic benefit, yet prospective controlled studies would be required to definitively mitigate baseline differences.

Our CGM analysis utilised the standard clinical 14‐day window rather than 28‐day data which may have been more robust [31]. However, the substantial sample size would have helped to mitigate individual inconsistencies. In our results, we noted no significant changes in eGFR, urinary ACR, or ALT. This may be attributed to the cohort's near‐normal baseline values and it remains unclear whether tirzepatide offers greater benefit in high‐risk populations with more prevalent established chronic kidney disease or MASLD. Finally, as this was a retrospective review, we lacked direct patient‐reported outcomes (PROs). Future research should formally evaluate how tirzepatide impacts the psychological burden of living with type 1 diabetes, particularly in terms of food noise and general well‐being.

## 6 Summary

As obesity rates rise among the type 1 diabetes population, there is an urgent need to address the disproportionate cardiovascular risk. While GLP‐1RAs are increasingly used off‐label to mitigate insulin resistance, regulatory approval has been hindered by a lack of dedicated outcome data. Our study provides robust real‐world evidence that tirzepatide delivers significant improvements in glycaemia, weight, and cardiometabolic risk markers, combined with a reassuring safety profile. These improvements were achieved without titration to maximum doses, which could represent a lower cost option with a lower burden of side effects. Large‐scale RCTs (such as the ongoing NCT06914895) are essential to confirm primary safety and efficacy, and future research must establish the long‐term durability of these benefits. Furthermore, it is critical to determine whether these benefits are limited to individuals with clinical obesity or extend to the broader type 1 diabetes population, and ultimately shift the focus from surrogate markers to definitive cardiovascular outcomes.

## Author Contributions

S.A.B. is the guarantor of the work and, as such, had full access to all the data in the study and takes responsibility for the integrity of the data and the accuracy of the data analysis. S.A.B. was involved in conception, design, data acquisition, analysis, interpretation of results, drafting the first draft, re‐drafting and approval of final version. E.M. and I.G. were involved in data acquisition, drafting the manuscript, and approval of the final version. J.E. and A.I. were involved in the conception, design, interpretation of results, drafting of the manuscript and approval of the final version.

## Funding

The authors have nothing to report.

## Conflicts of Interest

Simon A. Berry has received research support from Tandem Diabetes Care Inc. (San Diego, CA) and Dexcom Inc. (San Diego, CA). Has received travel grants through charities sponsored by Eli Lilly and Company (Indianapolis, IN) and Abbott UK (Maidenhead, UK). Jackie Elliott has received investigator‐led funding from Dexcom, and has received honoraria from Abbott, Boehringer, Dexcom, Eli Lilly, Glooko, Insulet, Medtronic, Novo Nordisk, Roche, Sanofi, and Ypsomed as advisory board or speaker fees. Ahmed Iqbal has received investigator‐led funding from Dexcom, Abbott, and has received honoraria from Eli Lilly, Sanofi, Boehringer Ingelheim, Ypsomed, and Diiachi Sankyo as speaker fees. Eleanor McNally and Iona R. Goodman have no conflicts of interest.

## Supporting information


**Table S1:** Table detailing concomitant anti‐cholesterol and antihypertensive medications at baseline.
**Table S2:** Between‐groups analysis of CGM metrics at end of follow‐up compared to baseline (Controls *n* = 41, tirzepatide *n* = 108). *p* values represent independent samples *t*‐tests (* *p* < 0.05, ** *p* < 0.01, *** *p* < 0.001), where non‐parametric distributions were observed (marked as †), Mann–Whitney U Tests were used in addition [square brackets]. Abbreviations: TBR—time below range; TITR—time in tight range; TIR—time in range; TAR—time above range; GMI—glycemic management index.
**Table S3:** Exploratory subgroup analysis comparing outcomes with tirzepatide in those on multiple dose injections (MDI) and hybrid closed loop (HCL). * *p* < 0.05, **p < 0.01, ***p < 0.001. Abbreviations: TDI—total daily dose of insulin, TBR—time below range, TITR—time in tight range, TIR—time in range, TAR—time above range, GMI—glycemic management index.
**Table S4:** Exploratory subgroup analysis comparing outcomes with tirzepatide and control in only those using hybrid closed loop (HCL) systems. * *p* < 0.05, **p < 0.01, ***p < 0.001. Abbreviations: TDI—total daily dose of insulin, TBR—time below range, TITR—time in tight range, TIR—time in range, TAR—time above range, GMI—glycemic management index.
**Table S5:** Exploratory subgroup analysis comparing outcomes in participants on tirzepatide with the Omnipod 5 (OP5) and Tandem T:Slim X2 hybrid closed loop systems. * *p* < 0.05, **p < 0.01, ***p < 0.001. Abbreviations: TDI—total daily dose of insulin, TBR—time below range, TITR—time in tight range, TIR—time in range, TAR—time above range, GMI—glycemic management index.
**Table S6:** Exploratory subgroup analysis comparing outcomes with tirzepatide in participants with a direct switch from another GLP‐1 receptor agonist and participants who were naïve to GLP‐1 receptor agonists at initiation. * *p* < 0.05, **p < 0.01, ***p < 0.001. Abbreviations: TDI—total daily dose of insulin, TBR—time below range, TITR—time in tight range, TIR—time in range, TAR—time above range, GMI—glycemic management index.

## Data Availability

The datasets generated during and/or analysed during the current study are not publicly available as they are collected from routine clinical care but anonymised data are available from the corresponding author upon reasonable request.
