# Supplementary material for: Real‐World Effectiveness and Safety of Tirzepatide in Type 1 Diabetes and Obesity: Impact on Glycaemia, Weight, and Cardiometabolic Risk Markers
Source: Diabetes Obes Metab. 2026 Jul 6;28(10):8836–46. doi: 10.1111/dom.71080 (PMC13538728; doi:10.1111/dom.71080)
Supplement: Supplementary file 1 [file DOM-28-8836-s001.docx]

*Supplementary Data*

## SUPPLEMENTARY TABLE 1

| Concomitant Medication | Controls N = 50^1^ | Tirzepatide  N = 142^1^ | Overall  N = 192^1^ |
| --- | --- | --- | --- |
| **Statin** | 27 (54.0%) | 86 (60.6%) | 113 (58.9%) |
| **Ezetimibe** | 5 (10.0%) | 7 (4.9%) | 12 (6.3%) |
| **Angiotensin-converting enzyme inhibitor** | 17 (34.0%) | 38 (26.8%) | 55 (28.6%) |
| **Angiotensin receptor blocker** | 4 (8.0%) | 15 (10.6%) | 19 (9.9%) |
| **Mineralocorticoid Receptor Agonist** | 1 (2.0%) | 3 (2.1%) | 4 (2.1%) |
| **Beta Blocker** | 3 (6.0%) | 19 (13.4%) | 22 (11.5%) |
| **Alpha Blocker** | 2 (4.0%) | 8 (5.6%) | 10 (5.2%) |
| **Nitrate** | 0 (0.0%) | 4 (2.8%) | 4 (2.1%) |
| **Loop Diuretic** | 1 (2.0%) | 10 (7.0%) | 11 (5.7%) |

## Supplementary Table 1: Table detailing concomitant anti-cholesterol and antihypertensive medications at baseline

## SUPPLEMENTARY TABLE 2

| Metric | Mean change (control) | Mean change (tirzepatide) | Mean difference | 95% CI | P value |
| --- | --- | --- | --- | --- | --- |
| TBR <3.0 mmol/L† | -0.17 | -0.07 | 0.10 | [-0.28, 0.47] | 0.603  [0.836] |
| TBR 3.0-3.9 mmol/L† | -0.17 | 0.19 | 0.37 | [-0.33, 1.06] | 0.299  [0.691] |
| TITR (3.9 – 7.8 mmol/L) | 0.49 | 5.68 | 5.19 | [1, 9.38] | **0.016*** |
| TIR (3.9-10.0 mmol/L) | 0.20 | 7.54 | 7.34 | [2.31, 12.38] | **0.005**** |
| TAR 10.0-13.9 mmol/L | 1.22 | -1.14 | -2.36 | [-5.27, 0.55] | 0.111 |
| TAR >13.9 mmol/L† | -1.07 | -6.52 | -5.45 | [-10.11, -0.78] | 0.022  [0.068] |
| Mean Sensor Glucose (mmol/L) | -0.09 | -0.86 | -0.77 | [-1.35, -0.2] | **0.009**** |
| GMI (mmol/mol) | -0.51 | -2.90 | -2.40 | [-4.81, 0.02] | 0.052 |
| Coefficient of Variation | -0.64 | -1.61 | -0.97 | [-3.07, 1.12] | 0.357 |
| Standard Deviation | -0.07 | -0.36 | -0.30 | [-0.6, 0.01] | 0.058 |

## Supplementary Table 2: Between-groups analysis of CGM metrics at end of follow-up compared to baseline (Controls n=41, tirzepatide n=108). P values represent independent samples t-tests (* p<0.05, ** p<0.01, *** p<0.001), where non-parametric distributions were observed (marked as †), Mann-Whitney U Tests were used in addition [square brackets]. Abbreviations: TBR – time below range; TITR – time in tight range; TIR – time in range; TAR – time above range; GMI – glycemic management index.

SUPPLEMENTARY TABLE 3

|  | Group | N | Baseline  Mean (SD) | End of Follow-Up  Mean (SD) | Mean Difference | 95% CI | p-value |
| --- | --- | --- | --- | --- | --- | --- | --- |
| HbA1c (mmol/mol)  HbA1c (%) | MDI | 54 | 67.8 (12.4)  8.4 (1.1) | 62.2 (12.0)  7.8 (3.2) | -5.7  -0.5 | [-8.3, -3.1]  [-0.8, 0.3] | **<0.001***** |
| Weight (kg) |  | 54 | 114.6 (21.9) | 102.03 (23.71) | -12.57 | [-15.31, -9.83] | **<0.001***** |
| TDI (units) |  | 15 | 142.79 (65.51) | 100.21 (75.54) | -42.59 | [-67.04, -18.14] | **0.002**** |
| Total Daily Basal (units) |  | 49 | 60.9 (36.81) | 47.85 (34.96) | -13.04 | [-18.31, -7.78] | **<0.001***** |
| Total Daily Bolus (units) |  | 14 | 80.71 (32.18) | 45.11 (31.85) | -35.60 | [-55.54, -15.66] | **0.002**** |
| TBR <3.0 mmol/L |  | 50 | 0.2 (0.57) | 0.1 (0.3) | -0.10 | [-0.26, 0.06] | 0.229 |
| TBR 3.0-3.9 mmol/L |  | 50 | 1.08 (2.19) | 1.68 (2.76) | 0.60 | [-0.34, 1.54] | 0.207 |
| TITR (3.9-7.8 mmol/L) |  | 50 | 21.78 (14.65) | 28.6 (20.15) | 6.82 | [0.92, 12.72] | **0.024*** |
| TIR (3.9-10.0 mmol/L) |  | 50 | 40.22 (20.01) | 48.16 (22.05) | 7.94 | [1.45, 14.43] | **0.017*** |
| TAR (10.0-13.9 mmol/L) |  | 50 | 28.62 (10.74) | 28.66 (12.22) | 0.04 | [-3.14, 3.22] | 0.980 |
| TAR (>13.9 mmol/L) |  | 50 | 29.88 (23.73) | 21.4 (19.69) | -8.48 | [-15.55, -1.41] | **0.020*** |
| Mean Sensor Glucose (mmol/L) |  | 50 | 11.71 (2.77) | 10.66 (2.67) | -1.05 | [-1.89, -0.21] | **0.016*** |
| GMI (mmol/mol) |  | 48 | 66.18 (10.87) | 63.61 (12.16) | -2.57 | [-5.34, 0.21] | 0.069 |
| Coefficient of Variation (%) |  | 48 | 34.09 (6.5) | 33.05 (6.64) | -1.04 | [-2.67, 0.6] | 0.208 |
| Standard Deviation |  | 48 | 3.87 (0.99) | 3.56 (1) | -0.30 | [-0.51, -0.1] | **0.005**** |
| HbA1c (mmol/mol)  HbA1c (%) | HCL | 54 | 60.5 (10.4)  7.7 (1.0) | 53.2 (8.1)  7.0 (0.7) | -7.3  -0.7 | [-9.3, -5.3]  [-0.9, -0.5] | **<0.001***** |
| Weight (kg) |  | 54 | 103.74 (18.1) | 93.08 (20.44) | -10.66 | [-13.07, -8.25] | **<0.001***** |
| TDI (units) |  | 52 | 81.18 (55.5) | 58.27 (40.63) | -22.90 | [-31.49, -14.32] | **<0.001***** |
| Total Daily Basal (units) |  | 52 | 51.5 (38.01) | 36.73 (26.51) | -14.78 | [-22.25, -7.3] | **<0.001***** |
| Total Daily Bolus (units) |  | 52 | 29.68 (20.36) | 20.77 (18.08) | -8.92 | [-11.52, -6.32] | **<0.001***** |
| TBR <3.0 mmol/L |  | 54 | 0.13 (0.39) | 0.09 (0.35) | -0.04 | [-0.17, 0.09] | 0.569 |
| TBR 3.0-3.9 mmol/L |  | 54 | 0.93 (1.2) | 0.85 (1.02) | -0.07 | [-0.45, 0.3] | 0.693 |
| TITR (3.9-7.8 mmol/L) |  | 54 | 32.44 (13.49) | 36.7 (15.75) | 4.26 | [0.65, 7.87] | **0.022*** |
| TIR (3.9-10.0 mmol/L) |  | 54 | 57.22 (14.64) | 64 (16.14) | 6.78 | [3.1, 10.46] | **<0.001***** |
| TAR (10.0-13.9 mmol/L) |  | 54 | 25.09 (6.96) | 22.52 (8.34) | -2.57 | [-4.52, -0.63] | **0.010*** |
| TAR (>13.9 mmol/L) |  | 54 | 16.63 (12.55) | 12.54 (11.45) | -4.09 | [-6.88, -1.3] | **0.005**** |
| Mean Sensor Glucose (mmol/L) |  | 54 | 10.14 (1.62) | 9.56 (1.56) | -0.57 | [-0.94, -0.21] | **0.003**** |
| GMI (mmol/mol) |  | 54 | 60.36 (7.66) | 57.66 (7.25) | -2.69 | [-4.47, -0.91] | **0.004**** |
| Coefficient of Variation (%) |  | 54 | 35.8 (5.68) | 33.47 (6.42) | -2.33 | [-3.92, -0.74] | **0.005**** |
| Standard Deviation |  | 54 | 3.65 (0.91) | 3.25 (1.02) | -0.40 | [-0.6, -0.2] | **<0.001***** |

Supplementary Table 3: Exploratory subgroup analysis comparing outcomes with tirzepatide in those on multiple dose injections (MDI) and hybrid closed loop (HCL). * p<0.05, **p<0.01, ***p<0.001. Abbreviations: TDI – total daily dose of insulin, TBR – time below range, TITR – time in tight range, TIR – time in range, TAR – time above range, GMI – glycemic management index.

SUPPLEMENTARY TABLE 4

|  | Treatment | N | Baseline  Mean (SD) | End of Follow-Up  Mean (SD) | Mean Difference | 95% CI | p-value |
| --- | --- | --- | --- | --- | --- | --- | --- |
| HbA1c (mmol/mol)  HbA1c (%) | Tirzepatide | 54 | 60.5 (10.4)  7.7 (1.0) | 53.24 (8.1)  7.0 (0.7) | -7.26  -0.7 | [-9.3, -5.3]  [-0.9, -0.5] | **<0.001***** |
| Weight (kg) |  | 54 | 103.74 (18.1) | 93.08 (20.44) | -10.66 | [-13.07, -8.25] | **<0.001***** |
| TDI (units) |  | 52 | 81.18 (55.5) | 58.27 (40.63) | -22.90 | [-31.5, -14.3] | **<0.001***** |
| Total Daily Basal (units) |  | 52 | 51.5 (38.01) | 36.73 (26.51) | -14.78 | [-22.25, -7.3] | **<0.001***** |
| Total Daily Bolus (units) |  | 52 | 29.68 (20.36) | 20.77 (18.08) | -8.92 | [-11.52, -6.32] | **<0.001***** |
| TBR <3.0 mmol/L |  | 54 | 0.13 (0.39) | 0.09 (0.35) | -0.04 | [-0.17, 0.09] | 0.569 |
| TBR 3.0-3.9 mmol/L |  | 54 | 0.93 (1.2) | 0.85 (1.02) | -0.07 | [-0.45, 0.3] | 0.693 |
| TITR (3.9-7.8 mmol/L) |  | 54 | 32.44 (13.49) | 36.7 (15.75) | 4.26 | [0.65, 7.87] | 0.022* |
| TIR (3.9-10.0 mmol/L) |  | 54 | 57.22 (14.64) | 64 (16.14) | 6.78 | [3.1, 10.46] | **<0.001***** |
| TAR (10.0-13.9 mmol/L) |  | 54 | 25.09 (6.96) | 22.52 (8.34) | -2.57 | [-4.52, -0.63] | **0.010*** |
| TAR (>13.9 mmol/L) |  | 54 | 16.63 (12.55) | 12.54 (11.45) | -4.09 | [-6.88, -1.3] | **0.005***** |
| Mean Sensor Glucose (mmol/L) |  | 54 | 10.14 (1.62) | 9.56 (1.56) | -0.57 | [-0.94, -0.21] | **0.003**** |
| GMI (mmol/mol) |  | 54 | 60.36 (7.66) | 57.66 (7.25) | -2.69 | [-4.47, -0.91] | **0.004**** |
| Coefficient of Variation (%) |  | 54 | 35.8 (5.68) | 33.47 (6.42) | -2.33 | [-3.92, -0.74] | **0.005**** |
| Standard Deviation |  | 54 | 3.65 (0.91) | 3.25 (1.02) | -0.40 | [-0.6, -0.2] | **<0.001***** |
| HbA1c (mmol/mol)  HbA1c (%) | Control | 15 | 57.5 (9.4)  7.4 (0.9) | 57 (7.3)  7.4 (0.7) | -0.47  -0.1 | [-4.8, 3.9]  [-0.4, 0.4] | 0.821 |
| Weight (kg) |  | 15 | 99.67 (14.11) | 104.6 (15.14) | 4.93 | [1.67, 8.2] | **0.006**** |
| TDI (units) |  | 14 | 63.36 (48.33) | 67.19 (41.43) | 3.84 | [-7.0, 14.7] | 0.460 |
| Total Daily Basal (units) |  | 14 | 35.09 (32.3) | 42.51 (32.7) | 7.43 | [-0.2, 15.06] | 0.056 |
| Total Daily Bolus (units) |  | 14 | 28.3 (19.6) | 24.7 (19.9) | -3.61 | [-11.3, 4.0] | 0.326 |
| TBR <3.0 mmol/L |  | 15 | 0.13 (0.35) | 0.07 (0.26) | -0.07 | [-0.32, 0.19] | 0.582 |
| TBR 3.0-3.9 mmol/L |  | 15 | 1.13 (1.36) | 1.13 (1.25) | 0.00 | [-0.55, 0.55] | 1.000 |
| TITR (3.9-7.8 mmol/L) |  | 15 | 35.2 (11.63) | 36.6 (9.06) | 1.40 | [-4.11, 6.91] | 0.594 |
| TIR (3.9-10.0 mmol/L) |  | 15 | 60.67 (11.33) | 60.73 (11.74) | 0.07 | [-6.61, 6.74] | 0.983 |
| TAR (10.0-13.9 mmol/L) |  | 15 | 24 (6.16) | 25.2 (4.81) | 1.20 | [-2.18, 4.58] | 0.459 |
| TAR (>13.9 mmol/L) |  | 15 | 14.07 (8.74) | 12.87 (8.72) | -1.20 | [-5.71, 3.31] | 0.577 |
| Mean Sensor Glucose (mmol/L) |  | 15 | 9.67 (1.17) | 9.61 (1.04) | -0.06 | [-0.7, 0.58] | 0.844 |
| GMI (mmol/mol) |  | 15 | 58.29 (5.52) | 57.83 (4.96) | -0.46 | [-3.47, 2.55] | 0.748 |
| Coefficient of Variation (%) |  | 15 | 36.73 (4.83) | 35.18 (5.26) | -1.55 | [-4.48, 1.39] | 0.277 |
| Standard Deviation |  | 15 | 3.57 (0.75) | 3.41 (0.79) | -0.16 | [-0.56, 0.25] | 0.428 |

Supplementary Table 4: Exploratory subgroup analysis comparing outcomes with tirzepatide and control in only those using hybrid closed loop (HCL) systems. * p<0.05, **p<0.01, ***p<0.001. Abbreviations: TDI – total daily dose of insulin, TBR – time below range, TITR – time in tight range, TIR – time in range, TAR – time above range, GMI – glycemic management index.

SUPPLEMENTARY TABLE 5

| Variable | Group | N | Baseline Mean (SD) | Follow-up Mean (SD) | Mean Difference | 95% CI | p-value |
| --- | --- | --- | --- | --- | --- | --- | --- |
| HbA1c (mmol/mol) | OP5 | 30 | 62.37 (11.82) | 54.4 (7.01) | -7.97 | [-11.19, -4.74] | **<0.001***** |
| Weight (kg) |  | 31 | 100.75 (16.65) | 90.02 (19.9) | -10.73 | [-14.3, -7.15] | **<0.001***** |
| TDI (units) |  | 31 | 71.8 (29.4) | 51.79 (31.7) | -20.01 | [-27.19, -12.82] | **<0.001***** |
| Total Daily Basal (units) |  | 31 | 46.5 (23.45) | 33.78 (25.65) | -12.72 | [-20.08, -5.37] | **0.001**** |
| Total Daily Bolus (units) |  | 31 | 25.3 (11.32) | 16.69 (9.26) | -8.61 | [-11.67, -5.55] | **<0.001***** |
| TBR <3.0 mmol/L |  | 31 | 0.1 (0.3) | 0.06 (0.25) | -0.03 | [-0.18, 0.12] | 0.662 |
| TBR 3.0-3.9 mmol/L |  | 31 | 0.84 (1.27) | 0.9 (1.11) | 0.06 | [-0.54, 0.67] | 0.829 |
| TITR (3.9-7.8 mmol/L) |  | 31 | 30.71 (13.38) | 34.03 (13.32) | 3.32 | [-2.03, 8.67] | 0.214 |
| TIR (3.9-10.0 mmol/L) |  | 31 | 54.06 (15.15) | 61.23 (15.18) | 7.16 | [1.92, 12.4] | **0.009**** |
| TAR (10.0-13.9 mmol/L) |  | 31 | 25 (6.51) | 23.65 (7.69) | -1.35 | [-3.99, 1.28] | 0.302 |
| TAR (>13.9 mmol/L) |  | 31 | 20 (14.47) | 14.16 (11.11) | -5.84 | [-9.73, -1.94] | **0.005**** |
| Mean Sensor Glucose (mmol/L) |  | 31 | 10.5 (1.79) | 9.8 (1.4) | -0.70 | [-1.26, -0.14] | **0.016*** |
| GMI (mmol/mol) |  | 31 | 62.33 (8.43) | 58.88 (6.64) | -3.44 | [-6.08, -0.81] | **0.012*** |
| Coefficient of Variation (%) |  | 31 | 36.29 (5.4) | 34.26 (6.13) | -2.03 | [-4.18, 0.13] | 0.064 |
| Standard Deviation |  | 31 | 3.83 (0.93) | 3.4 (0.96) | -0.43 | [-0.69, -0.16] | **0.003**** |
| HbA1c (mmol/mol) | T:slim X2 | 22 | 57.64 (7.63) | 50.73 (8.88) | -6.91 | [-9.07, -4.75] | **<0.001***** |
| Weight (kg) |  | 21 | 107.85 (20.38) | 96.94 (21.78) | -10.91 | [-14.52, -7.3] | **<0.001***** |
| TDI (units) |  | 20 | 94.65 (80.9) | 66.04 (51.01) | -28.61 | [-48.84, -8.37] | **0.008**** |
| Total Daily Basal (units) |  | 20 | 58.34 (53.88) | 40.15 (28.17) | -18.20 | [-34.94, -1.45] | **0.035*** |
| Total Daily Bolus (units) |  | 20 | 36.31 (28.84) | 25.91 (25.64) | -10.41 | [-15.08, -5.74] | **<0.001***** |
| TBR <3.0 mmol/L |  | 21 | 0.19 (0.51) | 0.14 (0.48) | -0.05 | [-0.32, 0.22] | 0.715 |
| TBR 3.0-3.9 mmol/L |  | 21 | 1 (1.14) | 0.81 (0.93) | -0.19 | [-0.59, 0.21] | 0.329 |
| TITR (3.9-7.8 mmol/L) |  | 21 | 35.05 (14.13) | 41.71 (18.23) | 6.67 | [1.59, 11.74] | **0.013*** |
| TIR (3.9-10.0 mmol/L) |  | 21 | 62.52 (13.11) | 70.62 (14.49) | 8.10 | [2.86, 13.33] | **0.004**** |
| TAR (10.0-13.9 mmol/L) |  | 21 | 25.24 (8.05) | 20.29 (8.97) | -4.95 | [-7.9, -2.01] | **0.002**** |
| TAR (>13.9 mmol/L) |  | 21 | 11.05 (6.72) | 8.14 (8.36) | -2.90 | [-6.75, 0.94] | 0.131 |
| Mean Sensor Glucose (mmol/L) |  | 21 | 9.54 (1.22) | 8.96 (1.46) | -0.58 | [-1, -0.17] | **0.008**** |
| GMI (mmol/mol) |  | 21 | 56.9 (5.19) | 54.7 (6.44) | -2.21 | [-4.55, 0.13] | 0.062 |
| Coefficient of Variation (%) |  | 21 | 34.5 (5.96) | 32 (6.95) | -2.50 | [-5.28, 0.29] | 0.076 |
| Standard Deviation |  | 21 | 3.31 (0.79) | 2.92 (1.01) | -0.39 | [-0.74, -0.03] | **0.035*** |

Supplementary Table 5: Exploratory subgroup analysis comparing outcomes in participants on tirzepatide with the Omnipod 5 (OP5) and Tandem T:Slim X2 hybrid closed loop systems. * p<0.05, **p<0.01, ***p<0.001. Abbreviations: TDI – total daily dose of insulin, TBR – time below range, TITR – time in tight range, TIR – time in range, TAR – time above range, GMI – glycemic management index.

SUPPLEMENTARY TABLE 6

|  | Group | N | Baseline  Mean (SD) | End of Follow-Up  Mean (SD) | Mean Difference | 95% CI | p-value |
| --- | --- | --- | --- | --- | --- | --- | --- |
| HbA1c (mmol/mol)  HbA1c (%) | Direct GLP-1 Switch | 25 | 65.0 (10.5)  8.1 (1.0) | 58.6 (8.0)  7.5 (0.7) | -6.3  -0.6 | [-10.3, -2.3]  [-0.9, -0.2] | **0.003**** |
| Weight (kg) |  | 27 | 110.54 (22.11) | 102.57 (23.08) | -7.97 | [-11.31, -4.63] | **<0.001***** |
| TDI (units) |  | 17 | 111.5 (96.06) | 88.41 (79.89) | -23.09 | [-48.13, 1.94] | 0.068 |
| Total Daily Basal (units) |  | 23 | 65.4 (58.49) | 52 (45.58) | -13.40 | [-28.26, 1.46] | 0.075 |
| Total Daily Bolus (units) |  | 16 | 44.11 (34.86) | 35.62 (33.85) | -8.49 | [-15.16, -1.81] | **0.016*** |
| TBR <3.0 mmol/L |  | 25 | 0.24 (0.6) | 0.16 (0.47) | -0.08 | [-0.32, 0.16] | 0.491 |
| TBR 3.0-3.9 mmol/L |  | 25 | 0.84 (1.55) | 0.72 (1.37) | -0.12 | [-0.63, 0.39] | 0.632 |
| TITR (3.9-7.8 mmol/L) |  | 25 | 25.72 (13.86) | 25.68 (14.26) | -0.04 | [-5.12, 5.04] | 0.987 |
| TIR (3.9-10.0 mmol/L) |  | 25 | 46.68 (18.67) | 49.04 (19.07) | 2.36 | [-5.3, 10.02] | 0.531 |
| TAR (10.0-13.9 mmol/L) |  | 25 | 26.56 (9.77) | 29 (8.74) | 2.44 | [-2.19, 7.07] | 0.287 |
| TAR (>13.9 mmol/L) |  | 25 | 25.68 (22.89) | 21.08 (16.83) | -4.60 | [-15.02, 5.82] | 0.371 |
| Mean Sensor Glucose (mmol/L) |  | 25 | 11.28 (2.84) | 10.75 (1.91) | -0.53 | [-1.69, 0.63] | 0.358 |
| GMI (mmol/mol) |  | 24 | 64.1 (10.07) | 63.59 (9.13) | -0.50 | [-4.24, 3.24] | 0.783 |
| Coefficient of Variation (%) |  | 24 | 35.25 (4.84) | 33.76 (5.28) | -1.50 | [-3.87, 0.88] | 0.205 |
| Standard Deviation |  | 24 | 3.83 (0.86) | 3.65 (0.89) | -0.17 | [-0.5, 0.16] | 0.295 |
| HbA1c (mmol/mol)  HbA1c (%) | GLP-1 Naive | 85 | 63.8 (12.3)  8.0 (1.1) | 57.5 (11.9)  7.4 (1.1) | -6.3  -0.6 | [-8.0, -4.6]  [-0.7, -0.4] | **<0.001***** |
| Weight (kg) |  | 85 | 108.8 (20.51) | 96.11 (22.43) | -12.70 | [-14.72, -10.67] | **<0.001***** |
| TDI (units) |  | 59 | 85.44 (44.9) | 60.57 (38.37) | -24.87 | [-33.13, -16.62] | **<0.001***** |
| Total Daily Basal (units) |  | 81 | 52.42 (28.75) | 38.92 (25.07) | -13.49 | [-17.57, -9.41] | **<0.001***** |
| Total Daily Bolus (units) |  | 53 | 39.51 (29.58) | 23.3 (18.33) | -16.21 | [-22.29, -10.13] | **<0.001***** |
| TBR <3.0 mmol/L |  | 83 | 0.17 (0.49) | 0.1 (0.3) | -0.07 | [-0.18, 0.04] | 0.203 |
| TBR 3.0-3.9 mmol/L |  | 83 | 1.13 (1.89) | 1.42 (2.19) | 0.29 | [-0.32, 0.89] | 0.344 |
| TITR (3.9-7.8 mmol/L) |  | 83 | 28.07 (15.77) | 35.47 (18.92) | 7.40 | [3.48, 11.32] | **<0.001***** |
| TIR (3.9-10.0 mmol/L) |  | 83 | 49.8 (20.31) | 58.89 (20.94) | 9.10 | [5.12, 13.07] | **<0.001***** |
| TAR (10.0-13.9 mmol/L) |  | 83 | 26.41 (9.13) | 24.19 (10.98) | -2.22 | [-4.1, -0.33] | **0.022*** |
| TAR (>13.9 mmol/L) |  | 83 | 22.49 (19.8) | 15.4 (16.29) | -7.10 | [-10.72, -3.48] | **<0.001***** |
| Mean Sensor Glucose (mmol/L) |  | 83 | 10.82 (2.36) | 9.85 (2.29) | -0.96 | [-1.43, -0.5] | **<0.001***** |
| GMI (mmol/mol) |  | 82 | 62.93 (10.49) | 59.33 (10.47) | -3.60 | [-5.38, -1.83] | **<0.001***** |
| Coefficient of Variation (%) |  | 82 | 34.83 (6.37) | 33.18 (6.71) | -1.65 | [-2.91, -0.38] | **0.011*** |
| Standard Deviation |  | 82 | 3.74 (1.01) | 3.31 (1.04) | -0.42 | [-0.57, -0.27] | **<0.001***** |

Supplementary Table 6: Exploratory subgroup analysis comparing outcomes with tirzepatide in participants with a direct switch from another GLP-1 receptor agonist and participants who were naïve to GLP-1 receptor agonists at initiation. * p<0.05, **p<0.01, ***p<0.001. Abbreviations: TDI – total daily dose of insulin, TBR – time below range, TITR – time in tight range, TIR – time in range, TAR – time above range, GMI – glycemic management index.
